# Contribution of Pretomanid to Novel Regimens Containing Bedaquiline with either Linezolid or Moxifloxacin and Pyrazinamide in Murine Models of Tuberculosis

**DOI:** 10.1128/AAC.00021-19

**Published:** 2019-04-25

**Authors:** Jian Xu, Si-Yang Li, Deepak V. Almeida, Rokeya Tasneen, Kala Barnes-Boyle, Paul J. Converse, Anna M. Upton, Khisimuzi Mdluli, Nader Fotouhi, Eric L. Nuermberger

**Affiliations:** a Center for Tuberculosis Research, Department of Medicine, Johns Hopkins University School of Medicine, Baltimore, Maryland, USA; b Beijing Key Laboratory of Drug Resistance Tuberculosis Research, Beijing Tuberculosis and Thoracic Tumor Research Institute, and Beijing Chest Hospital, Capital Medical University, Beijing, China; c Global Alliance for Tuberculosis Drug Development, New York, New York, USA; d Department of International Health, Johns Hopkins Bloomberg School of Public Health, Baltimore, Maryland, USA

**Keywords:** bedaquiline, linezolid, moxifloxacin, murine model, pretomanid, pyrazinamide, resistance

## Abstract

Novel regimens combining bedaquiline and pretomanid with either linezolid (BPaL regimen) or moxifloxacin and pyrazinamide (BPaMZ regimen) shorten the treatment duration needed to cure tuberculosis (TB) in BALB/c mice compared to that of the first-line regimen and have yielded promising results in initial clinical trials. However, the independent contribution of the investigational new drug pretomanid to the efficacy of BPaMZ has not been examined, and its contribution to BPaL has been examined only over the first 2 months of treatment. In the present study, the addition of pretomanid to BL increased bactericidal activity, prevented emergence of bedaquiline resistance, and shortened the duration needed to prevent relapse with drug-susceptible isolates by at least 2 months in BALB/c mice. Addition of pretomanid to bedaquiline, moxifloxacin, and pyrazinamide (BMZ) resulted in a 1-log_10_ greater CFU reduction after 1 month of treatment and/or reduced the number of mice relapsing in each of 2 experiments in BALB/c mice and in immunocompromised nude mice. Bedaquiline-resistant isolates were found at relapse in only one BMZ-treated nude mouse. Treatment of infection with a pyrazinamide-resistant mutant in BALB/c mice with BPaMZ prevented selection of bedaquiline-resistant mutants and reduced the proportion of mice relapsing compared to that for BMZ treatment alone. Among severely ill C3HeB/FeJ mice with caseous pneumonia and cavitation, BPaMZ increased median survival (≥60 versus 21 days) and reduced median lung CFU by 2.4 log_10_ at 1 month compared to the level for BMZ. In conclusion, in 3 different mouse models, pretomanid contributed significantly to the efficacy of the BPaMZ and BPaL regimens, including restricting the selection of bedaquiline-resistant mutants.

## Abstract (precis)

Novel regimens combining bedaquiline and pretomanid with either linezolid (BPaL regimen) or moxifloxacin and pyrazinamide (BPaMZ regimen) shorten the treatment duration needed to cure tuberculosis (TB) in BALB/c mice compared to that of the first-line regimen and have yielded promising results in initial clinical trials. However, the independent contribution of the investigational new drug pretomanid to the efficacy of BPaMZ has not been examined, and its contribution to BPaL has been examined only over the first 2 months of treatment.

## INTRODUCTION

The World Health Organization (WHO) estimates that 10.4 million people developed active tuberculosis (TB) in 2016, and 1.67 million people died from it (1). Nearly 500,000 new cases of multidrug-resistant TB (MDR-TB) occur annually, with an estimated treatment success rate of only 54% (1, 2). The current standard short-course regimen for drug-susceptible TB, consisting of rifampin (RIF), isoniazid (INH), pyrazinamide (PZA), and ethambutol (EMB) (regimen abbreviated as RHZE), requires a 6-month treatment duration to provide sufficient population-level efficacy. It takes 9 to 24 months for regimens containing at least 4 to 6 drugs, including at least one injectable agent, to treat patients with MDR-TB (3). New regimens to shorten and simplify TB treatment are urgently needed. If such regimens do not contain INH or RIF, they may be applicable to both drug-susceptible TB and MDR-TB.

The combination of bedaquiline (BDQ), pretomanid (PMD), moxifloxacin (MXF), and PZA (regimen abbreviated as BPaMZ) had bactericidal and sterilizing activity superior to that of RIF+INH+PZA in a murine model of TB, shortening the duration of treatment required to prevent relapse by 2.5 to 3.5 months (4). In the subsequent phase 2 NC-005 trial (ClinicalTrials registration no. NCT02193776), PZA-susceptible MDR-TB patients receiving the BPaMZ regimen had significantly faster sputum culture conversion than drug-susceptible TB patients receiving RIF+INH+PZA+EMB (5), showing that mouse data were predictive for the clinical trial results for the regimens tested. A phase 3 trial evaluating the BPaMZ regimen administered for 4 months in drug-susceptible TB patients and for 6 months in MDR-TB patients is now enrolling subjects (ClinicalTrials registration no. NCT03338621). The combination of BDQ, PMD, and linezolid (LZD) (regimen abbreviated as BPaL) also has superior bactericidal and sterilizing activity compared to that of RHZE in a murine TB model (6). Although it does not cure mice as rapidly as BPaMZ, this regimen has a greater spectrum of activity and has recently shown promising efficacy as an all-oral 6-month regimen in patients (Nix-TB trial) with extensively drug-resistant TB (7).

It is important to understand the contribution of each component in a regimen that is moving forward in the clinic. The independent contributions of BDQ, MXF, and PZA to the efficacy of BPaMZ were previously demonstrated in a BALB/c mouse TB model (4). Furthermore, receipt of the BPaMZ regimen was associated with numerically higher sputum conversion rates in PZA-susceptible MDR-TB patients than in drug-susceptible TB patients receiving the BDQ+PMD+PZA regimen and PZA-resistant MDR-TB patients receiving BPaMZ in the NC-005 trial (5), indicating the contribution of MXF and PZA. In addition, the sputum conversion rates after 2 months of treatment with BPaMZ in the NC-005 trial were higher than those in MDR-TB patients receiving the same regimen without BDQ in the NC-002 trial (8), indicating the contribution of BDQ.

PMD is a nitroimidazole drug that is activated within Mycobacterium tuberculosis by the bacterial deazaflavin-dependent nitroreductase Ddn, and it has bactericidal activity against replicating and nonreplicating bacilli (9, 10). The contribution of this new investigational drug to the BPaMZ regimen has yet to be confirmed directly in preclinical or clinical studies. Indeed, addition of PMD antagonized the bactericidal activity of BDQ, BDQ+PZA, and BDQ+PZA+clofazimine (CFZ) in past experiments in mice (11–13). However, the addition of PMD increased the bactericidal activity when added to BDQ+LZD and increased both the bactericidal and sterilizing activity when added to BDQ+sutezolid (6, 12). Another possible advantage of including PMD in the BPaMZ regimen is that it could reduce the selection of BDQ-resistant mutants, since, considering the reliably active drugs remaining in the regimen, such mutants would be more effectively targeted by PaMZ (PMD+MXF+PZA) than by MZ (MXF+PZA) alone (since PaMZ is a synergistic combination [14]).

The present study was undertaken to confirm the independent contributions of PMD to BPaMZ and BPaL by assessing the efficacy of each regimen with and without inclusion of PMD. Both regimens were evaluated in the same high-dose aerosol infection model in BALB/c mice in which their therapeutic potential was first described (4, 6). The contribution of PMD to the 4-drug BPaMZ regimen was further evaluated in athymic nude mouse and C3HeB/FeJ mouse models of TB. Athymic nude mice, which lack mature, differentiated T cells and are thus deprived of cell-mediated immunity, are more prone to relapse and the emergence of drug-resistant mutants than BALB/c mice (15, 16), thereby providing a more stringent model for evaluating the ability of the regimen to truly sterilize the infection and/or prevent the selection of drug-resistant mutants. This model may be more representative of TB in patients with immunocompromising diseases such as human immunodeficiency virus (HIV) or following iatrogenic immunosuppression who have an increased risk of treatment failure and relapse, especially those not receiving antiretroviral therapy (17).

C3HeB/FeJ mice are increasingly being utilized for TB drug development because, unlike BALB/c mice, which develop only cellular granulomas following infection with M. tuberculosis, the former develop caseating necrotic lung lesions, including cavities, that more closely resemble the pathological hallmarks of human TB (18–21). The necrotic lesion can have a profound effect on drug efficacy by altering drug partitioning across the lesion into caseum, as well as by presenting different microenvironments in caseum which can lead to reduced drug activity (18, 19, 22–24). Therefore, comparative studies are useful to evaluate the potential effect of these pathological differences on drug efficacy.

## RESULTS

### Experiment 1: comparison of BPaMZ and BMZ in BALB/c and athymic nude mice.

The scheme of this experiment is shown in Table S1 in the supplemental material. The aerosol infection implanted nearly 4 log_10_ CFU in the lungs of both mouse strains (Table 1). At the start of the treatment 14 days postinfection, BALB/c mice and nude mice harbored approximately 7.95 and 7.56 log_10_ CFU in their lungs, respectively. After 1 month of treatment, the addition of PMD to bedaquiline, moxifloxacin, and pyrazinamide (BMZ) resulted in an additional reduction of approximately 1 log_10_ in both BALB/c and nude mice (*P* < 0.01). Both regimens resulted in significantly greater decreases in lung CFU counts in BALB/c mice compared to counts in nude mice, with additional 1.1-log_10_ reductions observed in BALB/c mice after 1 month of treatment with both BPaMZ and BMZ (*P* < 0.01). Based on prior experience (4), BALB/c mice were expected to be culture negative after 2 months of treatment and were not assessed at that time point. Among nude mice, all mice in the BPaMZ group and 7 of 10 mice in the BMZ group were culture negative at 2 months despite plating the entire lung homogenate. Only a few CFU were detected in the other 3 mice of the BMZ group.

**TABLE 1:** Lung CFU counts assessed during treatment and proportion of mice relapsing after treatment completion in experiment 1

Mouse strain and drug regimen	Mean (±SD) log_10_ CFU count at^*a*^:				Proportion (%) relapsing after treatment for:		
	D−13	D0	M1	M2	1.5 mo	2 mo	2.5 mo
BALB/c							
Untreated	3.98 ± 0.09	7.95 ± 0.25					
BPaMZ			0.53 ± 0.44		3/15 (20)	0/16 (0)	
BMZ			1.48 ± 0.28		2/15 (13)	0/15 (0)	
Nude							
Untreated	3.97 ± 0.09	7.56 ± 0.17					
BPaMZ			1.63 ± 0.49	0.00 ± 0.00			1/18 (6)
BMZ			2.62 ± 0.42	0.31 ± 0.61			4/16 (25)

a Time points are shown as days (D−13 or D0) or months (M1 or M2) of treatment. 1.5 mo indicates that the mice were held for 3 additional months after completing 1.5 months of treatment.

Relapse was assessed 3 months after completing 1.5 and 2 months of treatment in BALB/c mice and after 2.5 months of treatment in nude mice (Table 1). In BALB/c mice, no significant difference was observed in the proportions of mice relapsing after treatment with BPaMZ (3/15 [20%)] or BMZ (2/15 [13%]) for 1.5 months (*P* = 1.0). Both groups were relapse free after 2 months of treatment. Among nude mice, which required a longer duration of treatment to cure, the proportion relapsing after 2.5 months of treatment was higher in BMZ-treated mice (4/16 [25%]) than in BPaMZ-treated mice (1/18 [6%]), but the difference was not statistically significant (*P* = 0.16). Three nude mice in the BMZ group were euthanized when they became moribund just 6 weeks after completing treatment. Despite some colonies isolated from the lung homogenates having an atypical morphology, they were confirmed to be M. tuberculosis by acid-fast staining and 16S rRNA sequencing. Therefore, these mice were counted as relapses. Three nude mice in the BPaMZ group also required euthanasia when they became moribund 9 weeks after completing treatment. However, the lung homogenates from these 3 mice yielded no growth, except for 1 colony from the lungs of one mouse that was subsequently identified by colony morphotype, AFB staining, and 16S rRNA sequencing as Staphylococcus epidermidis. Therefore, these mice were not counted as relapses.

We hypothesized that the addition of PMD to BMZ would reduce the selection of BDQ-resistant mutants in nude mice. At the start of treatment, lung homogenates from 5 mice were plated in parallel on medium containing 0.06 μg/ml of BDQ or 2 μg/ml of PMD. The mean frequencies against BDQ and PMD were 1.3 × 10^−6^ and 6.1 × 10^−6^, respectively, compared to the total CFU counted on drug-free plates. Three to five individual BDQ-resistant colonies were selected from each mouse for sequencing of the *Rv0678*, *pepQ*, and *atpE* genes. Spontaneous *Rv0678* mutants were identified in all 5 mice, and unique *pepQ* mutants were also found in 2 of the 5 mice (Table S2). None of the 7 colonies tested had *atpE* mutations. In total, 15 unique mutations (most of them frameshift mutations) were scattered across the *Rv0678* gene. Two mutants isolated on BDQ-containing plates (colonies 8 and 16) were selected for whole-genome sequencing (WGS) to confirm the mutations. WGS confirmed the *Rv0678* mutations previously identified by PCR-based sequencing and the absence of other mutations. Among relapsing mice, a single colony grew on BDQ-containing plates from 1 of 3 nude mice relapsing 6 weeks after completing 10 weeks of BMZ treatment. It harbored a c313t (R105C) mutation in *Rv0678*. However, this mutant represented a very small proportion of the total CFU count, similar to the baseline frequency at D0, suggesting that it reflected a spontaneous mutation arising during multiplication after treatment ended. Growth amounting to more than 1% of the total CFU count was observed on BDQ-containing plates from one mouse relapsing at 12 weeks posttreatment with BMZ, and sequencing revealed a *pepQ* mutation (g896t), indicating that this mutant was selectively amplified during treatment. Among all the BDQ-resistant mutants, frameshift mutations in *Rv0678* were routinely associated with 2-fold-higher MICs than single-nucleotide polymorphisms (SNPs) in *Rv0678* and *pepQ* (Table 2).

**TABLE 2:** Lung CFU counts during treatment against M. tuberculosis H37Rv WT and *pncA* mutant and proportion of mice relapsing after treatment completion in experiment 2

Regimen	Mean lung log_10_ CFU count^*a*^ (±SD)				Proportion of mice relapsing after treatment for^*b*^:				
	D−14	D0	M1	M2	1 mo	1.5 mo	2 mo	3 mo	4 mo
WT									
Untreated	4.06 ± 0.05	7.90 ± 0.16							
BL			4.87 ± 0.16	2.69 ± 0.30			15/15^7^	15/15^5^	14/15^5^
BPaL			3.29 ± 0.09	0.68 ± 0.24			7/15	0/15	0/15
BMZ			1.29 ± 0.19		15/15	6/15	1/15		
BPaMZ			1.05 ± 0.18		14/15	0/15	0/15		
*pncA* mutant									
Untreated	4.36 ± 0.17	8.09 ± 0.08							
BMZ			4.06 ± 0.23	1.24 ± 0.17			15/15^3^	7/20^3^	
BPaMZ			4.22 ± 0.23	1.61 ± 0.32			15/15^1^	0/20	

a Time points are shown as days (D−14 or D0) or months (M1 or M2) of treatment. 1 mo indicates that the mice were held for 3 additional months after completing 1 month of treatment.

b Superscript numbers represent the number of mice with isolates resistant to 0.125 mg/liter BDQ.

### Experiment 2: confirmation of the contribution of PMD to the BPaMZ and BPaL regimens in BALB/c mice and evaluation of the impact of baseline PZA or PMD resistance.

**FIG 1:**
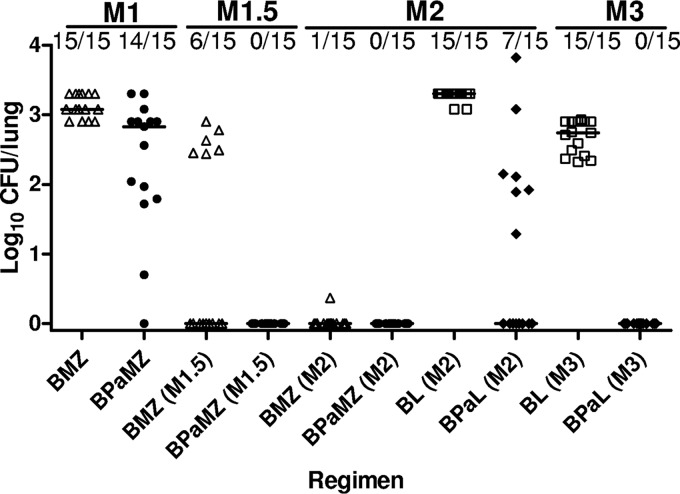
Proportion of relapses and individual mouse lung CFU counts after treatment of infection with M. tuberculosis H37Rv for 1 month (M1), 1.5 months (M1.5), 2 months (M2), and 3 months (M3) with each regimen. Regimen symbols: BMZ, open triangles; BPaMZ, solid circles; BL, open squares; and BPaL, solid diamonds. Horizontal black lines indicate the medians.

A second experiment was performed to confirm the results of experiment 1, to evaluate the contribution of PMD to BPaMZ in the event of baseline PZA resistance, to assess the contribution of PMD to the sterilizing activity of BPaL, and to confirm that the contribution of PMD to BPaMZ and BPaL is directly attributable to its antituberculosis activity upon activation by Ddn. The schemes for this experiment are shown in Tables S3 and S4. Mice were infected in parallel with the H37Rv strain or either of the isogenic PZA- or PMD-resistant mutants. Mean lung CFU counts exceeded 4 log_10_ CFU on the day after infection (Table 2). At the start of the treatment 2 weeks later, mean CFU counts were approximately 8 log_10_ CFU in the H37Rv and *pncA* mutant infection groups, respectively, and modestly lower in the *ddn* mutant group. Among mice infected with the H37Rv parent, the addition of PMD to BMZ did not result in a statistically significant decrease in lung CFU counts after 1 month of treatment. However, the addition of PMD was associated with fewer relapses after 1.5 months of treatment (*P* = 0.02), as well as lower CFU counts at the relapse assessments after 1 (*P* = 0.001) and 1.5 months of treatment (*P* = 0.02) (Fig. 1 and Table 2). The isolate from the mouse that relapsed after 2 months of BMZ treatment was not BDQ resistant.

**FIG 2:**
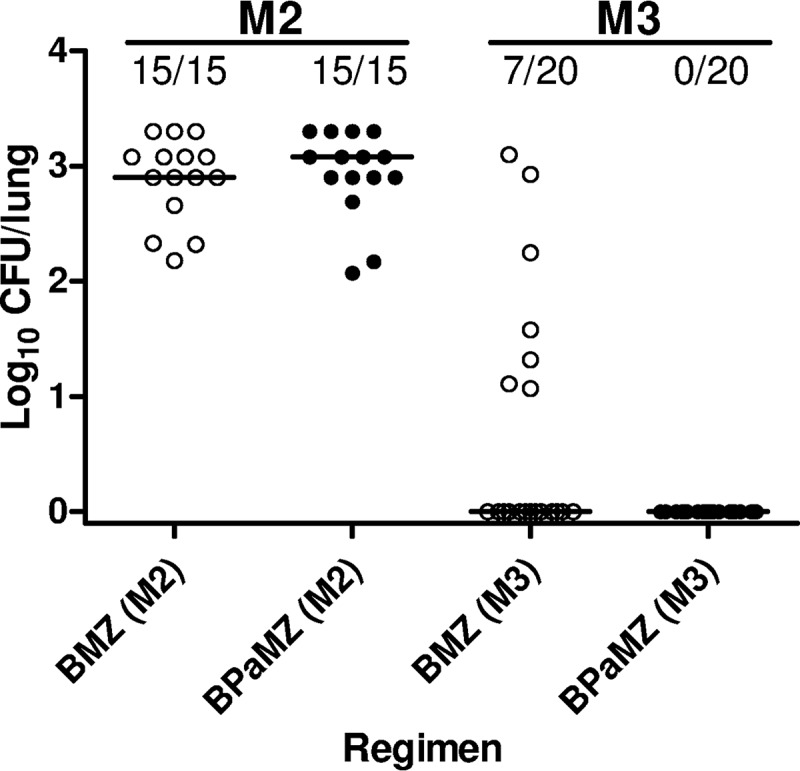
Proportion of relapses and individual mouse lung CFU counts (with medians) after treatment of infection with M. tuberculosis *pncA* A146V mutant for 2 months (M2) and 3 months (M3) with each regimen. BMZ, open black circles; BPaMZ, solid black circles.

As expected, both regimens were significantly less effective against the *pncA* mutant, consistent with the important contribution of PZA previously observed in wild-type (WT) infections (4). No significant effect of PMD was observed in the mean CFU counts after 1 or 2 months of treatment (Table 2). However, addition of PMD was associated with significantly fewer relapses (*P* = 0.01) (Table 2) and lower CFU counts at the relapse assessment (*P* = 0.01) after 3 months of treatment (Fig. 2). Addition of PMD also prevented the selection of BDQ-resistant mutants in *pncA* mutant-infected mice. Seven mice receiving BMZ (3 and 4 mice treated for 2 and 3 months, respectively) showed bacterial growth on agar plates containing BDQ at 0.125 μg/ml that exceeded 1% of the growth on drug-free plates (range, 15% to 100%) compared to growth for just one mouse receiving BPaMZ for 2 months (*P* = 0.05). Six of the 7 isolates tested from BDQ-containing plates had mutations in *Rv0678* (5) or *pepQ* (1) (Table 3). After 2 months of treatment in *pncA* mutant-infected mice, 3/15 relapses in the BMZ group had BDQ-resistant CFU with unique mutations of g362a and an a436 insertion in *Rv0678* and a g812 insertion in *pepQ,* whereas 1/15 relapses in the BPaMZ group had an a202g mutation in *Rv0678*. After 3 months of treatment, 3 relapses in the BMZ group were BDQ-resistant CFU with t407c substitution or a g168 deletion in *Rv0678* in 2 isolates and wild-type *Rv0678* and *pepQ* sequences.

**TABLE 3:** Mutations observed in M. tuberculosis colonies isolated from relapsing BALB/c mice on bedaquiline-containing plates in experiment 2

Time point	Strain background	Mouse no.	Sequence^*a*^	
			*Rv0678*	*pepQ*
M2 + 3	*pncA* mutant	BMZ-1	g362a (G120E)^2/2^	
		BMZ-11	a436 insertion (146 codon shift)^2/2^	
		BMZ-12	WT^2/2^	g812 insertion (271-codon shift)^2/2^
		BPaMZ-15	a202g (S68G)^2/2^	
M3 + 3	*pncA* mutant	BMZ-1	WT	WT
		BMZ-9	G deletion at nt 168 (56-codon shift)^2/2^	
		BMZ-13	t407c (L136P)^2/2^	
	WT	BL-4	g73t (G25C)	
		BL-6	WT	t68c (M23T)
		BL-7	WT	WT
		BL-11	t128c (L43P)	
		BL-14	g457c (A153P)	WT
M4 + 3	WT	BL-6	g320t (R107C)^1/2^, WT^1/2^	
		BL-8	g73t (G25C)^2/2^	
		BL-9	g457c (A153P)^2/2^	
		BL-12	g74a (G25D)^1/2^, g197a(G66E)^1/2^	
		BL-14	c286t(R96W)^1/2^, WT^1/2^	

a Superscript numbers indicate the proportion of colonies with the indicated genotype among the colonies tested.

Among mice infected with the H37Rv parent, addition of PMD to BL was associated with significantly lower mean CFU counts after 1 (*P* < 0.0001) and 2 (*P* = 0.0006) months of treatment (Table 2). Addition of PMD also had a marked effect on sterilizing activity. The proportion of mice relapsing after treatment with BPaL for just 2 months (7/15 [47%]) was lower than that observed in mice receiving BL for 4 months (14/15 [93%]) (*P* = 0.01), indicating that inclusion of PMD reduced the treatment duration necessary to prevent relapse by at least 2 months. Addition of PMD also prevented the selection of BDQ-resistant mutants. Seven, five, and five mice relapsing after receiving BL for 2, 3, and 4 months, respectively, had CFU growing on BDQ-containing plates exceeding 1% of the total CFU count, and an additional mouse in the 2-month treatment cohort barely missed this threshold. The actual proportions of CFU growing on BDQ increased with treatment duration (1% to 5%, 2% to 22%, and 2% to 41% after 2, 3, and 4 months of treatment, respectively). In contrast, no growth was observed on BDQ-containing plates in any mouse relapsing after BPaL treatment (*P* < 0.0001). Four of the 5 isolates from BDQ-containing plates at the relapse assessment 3 months after 3 months of treatment (M3 + 3) were tested and had mutations detected in *Rv0678* (3 isolates with g73t, t128c, or g457c substitutions) or *pepQ* (1 isolate with t68c substitution), while the remaining isolate had wild-type sequences in these genes (Table 3). All 5 mice relapsing at relapse assessment 3 months after 4 months of treatment harbored *Rv0678* mutants with single g320t, g73t, g457c, or c286t substitution or both g74a and g197a substitutions in 2 isolates from one mouse (Table 3).

As expected, infection with the PMD-resistant *ddn* mutant eliminated the contribution of PMD to both the BPaMZ and BPaL regimens (Table 4). In fact, a trend toward modest dose-dependent antagonism was observed in mean CFU count comparisons when adding PMD to these combinations.

**TABLE 4:** Lung CFU counts assessed during treatment against a *ddn* mutant and proportion of mice relapsing after treatment completion in experiment 2

Regimen	Mean lung log_10_ CFU count^*a*^ (±SD)				Proportion of mice relapsing after treatment for:		
	D−14	D0	M1	M2^*b*^	1 mo	2 mo	3 mo
Untreated	4.23 ± 0.07	7.61 ± 0.19					
Pa_50_			7.34 ± 0.12				
Pa_100_			7.26 ± 0.07				
BL			4.54 ± 0.17	2.97 ± 0.28			15/15
BPa_50_L			4.68 ± 0.41	3.05 ± 0.28			15/15
BPa_100_L			5.31 ± 0.35	3.33 ± 0.09			15/15
BMZ			2.36 ± 0.63	0.00 ± 0.00	15/15	2/15	
BPa_50_MZ			2.49 ± 0.24	0.08 ± 0.19	15/15	1/15	
BPa_100_MZ			2.73 ± 0.46	0.00 ± 0.00	15/15	1/15	

a Time points are shown as days (D−14 or D0) or months (M1 or M2) of treatment. 1 mo indicates that the mice were held for 3 additional months after completing 1 month of treatment.

b Five mice receiving pretomanid monotherapy at either 50 or 100 mg/kg (Pa_50_ or Pa_100_, respectively) became ill, were euthanized at week 1, and had a mean lung CFU count of 9.07 log_10_ CFU. One mouse in the BL group also died at week 1 due to a gavage accident. The lung contained 7.04 log_10_ CFU.

### Experiment 3: comparison of BPaMZ and BMZ in C3HeB/FeJ mice.

**FIG 3:**
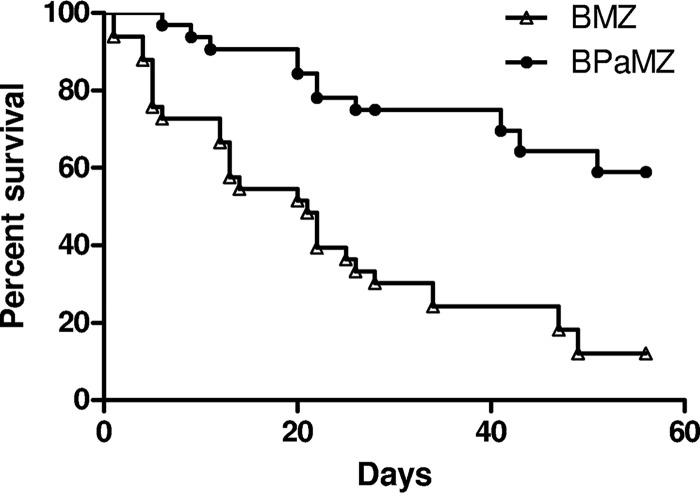
Survival of C3HeB/FeJ mice infected with M. tuberculosis HN878 from the onset of treatment with BMZ or BPaMZ.

**FIG 4:**
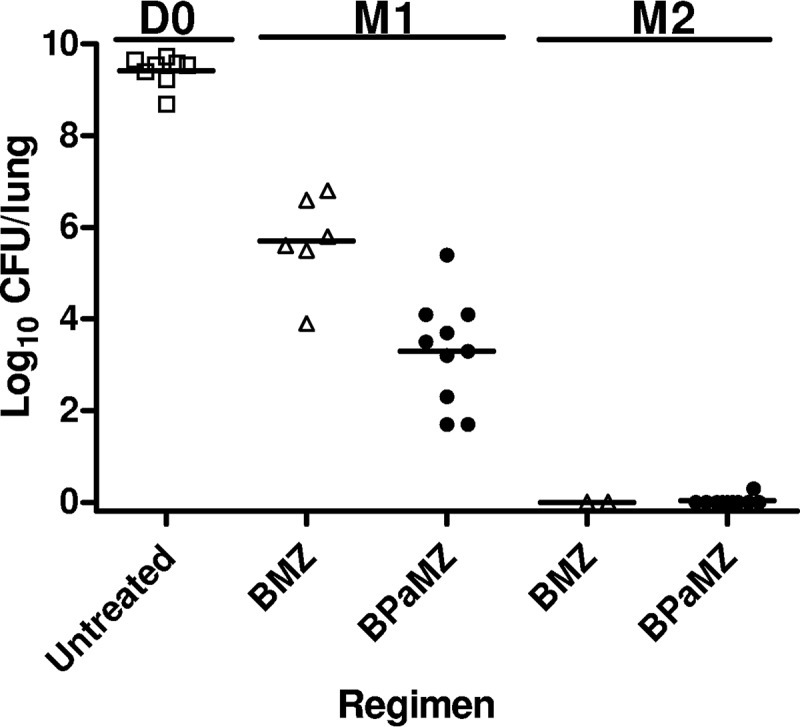
Lung CFU counts assessed during treatment in infected C3HeB/FeJ mice. Data points indicate individual mouse CFU counts. Horizontal black lines indicate the medians.

The scheme for experiment 3 is shown in Table S5. The two aerosol infections each implanted approximately 3 log_10_ CFU in the lungs of C3HeB/FeJ mice. By the start of treatment 4 weeks after the first infection, the mean CFU count had increased to 9.43 ± 0.33 log_10_. Due to the unexpectedly high burden of infection and the rapidly evolving lung damage under way at treatment onset, substantial mortality was observed over the ensuing 2-month treatment period despite the strong bactericidal effect of both regimens. Addition of PMD to the BMZ regimen extended the median survival from 21 days to more than 60 days (*P* < 0.0001) (Fig. 3) and significantly increased the bactericidal activity. After 1 month of treatment, the median lung CFU count was 2.4 log_10_ lower among mice receiving BPaMZ than those receiving BMZ (*P* < 0.01) (Fig. 4). After 2 months of treatment, only 2 BMZ-treated mice survived, whereas 10 BPaMZ-treated mice survived. Other than 1 BPaMZ-treated mouse with 2 CFU, all mice were culture negative. No colonies were isolated on plates containing BDQ (0.06 μg/ml) or PMD (2 μg/ml) at either time point.

**FIG 5:**
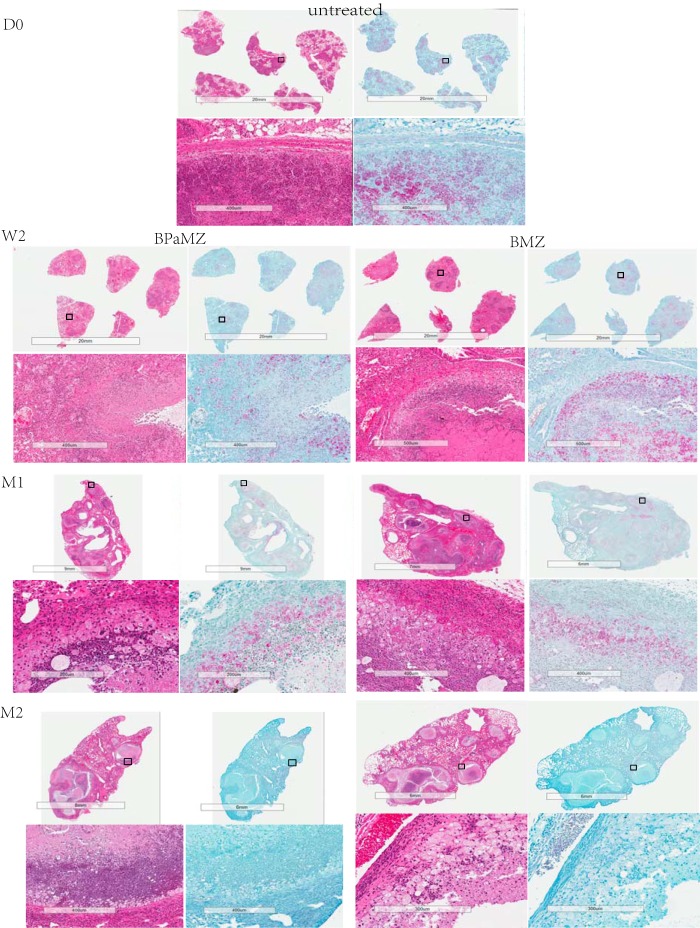
Lung histopathology in C3HeB/FeJ mice before and during treatment with BPaMZ (left) or BMZ (right) beginning 4 weeks postinfection with M. tuberculosis HN878. Hematoxylin and eosin (H&E) and Ziehl-Neelsen (AFB) staining were performed on lung tissue sections. Low-power views of an entire lung section (upper) and higher-power views of individual granulomas or cavitary lesions (lower) from representative mice in each group are shown. D0, treatment initiation (4 weeks postinfection); W2, status after 2 weeks of treatment; M1, status after 1 month of treatment; M2, status after 2 months of treatment.

At 4 weeks postinfection (D0) with a high aerosol dose of M. tuberculosis HN878 under an accelerated disease protocol, C3HeB/FeJ mice exhibited extensive lung involvement with both cellular and caseating lesions (Fig. 5). Cellular lesions were composed of neutrophilic clusters interspersed with lymphocytes and epithelioid macrophages. Caseating lesions included both isolated and coalescing granulomas with various degrees of central caseation and cellularity (Fig. 5, D0). Dense neutrophilic infiltration and abundant intracellular and extracellular acid-fast bacilli were evident at the foamy macrophage-caseum interface, whereas the proportion of extracellular bacilli was higher toward the more acellular center of caseous lesions (Fig. 5, D0, AFB staining). By 6 weeks postinfection (week 2 [W2] of treatment), the extent of lung disease had increased despite treatment. While some caseating lesions displayed more organized structures with an increasingly well-defined fibrous rim, more extensive central caseation, and even cavitation, other areas displayed extensive infiltration with exudative pneumonitis (Fig. 5, W2). Extracellular bacteria were increasingly evident in the acellular caseum (Fig. 5, W2, AFB staining). At 8 weeks postinfection (M1 of treatment), lung volumes were dominated by large areas of necrosis with central caseation and cavitary lesions (Fig. 5, M1). Multiple lesion types presented at this time, suggesting heterogeneous disease progression. After 1 month of treatment with BPaMZ or BMZ, acid-fast staining was more diffuse, possibly reflective of structural deterioration of bacteria by the highly bactericidal regimens (Fig. 5, M1, AFB staining). After 2 months of treatment, similar pathological changes were evident on H&E staining (Fig. 5, M2), but no visible intact acid-fast bacilli were observed in either treatment group (Fig. 5, M2, AFB staining).

## DISCUSSION

The BPaL and BPaMZ regimens have the potential to transform the treatment of both drug-susceptible and drug-resistant TB. BPaL shows promise as an oral short-course (e.g., 6-month) regimen for multidrug- and extensively drug-resistant TB (7). BPaMZ may be capable of shortening the treatment of drug-susceptible TB to 4 months or fewer and could reasonably be expected to do the same for multidrug-resistant TB with preserved susceptibility to MXF and PZA (5). Both regimens were identified in a comprehensive screening program seeking to identify broad-spectrum regimens containing two or more novel agents with minimal preexisting resistance in our high-dose aerosol infection model in BALB/c mice (4, 6). However, while the independent contributions of each other component of these regimens have been confirmed in this model (4, 6), the contribution of the new investigational drug PMD to the BPaMZ regimen was not previously demonstrated, and its contribution to the BPaL regimen was only assessed as far as the bactericidal activity of the regimen over the first 2 months of treatment and not for the sterilizing activity of the regimen.

The present study assessed the contribution of PMD to the BPaMZ regimen in 3 different murine models of TB and its contribution to the sterilizing activity of BPaL in BALB/c mice. Studies using the relapse endpoint in BALB/c mice have demonstrated utility for regimen selection and estimation of treatment duration (25, 26). The present results reinforce our prior findings that BPaMZ has remarkable bactericidal and sterilizing activity in BALB/c mice (4) and extend them by demonstrating the independent contribution of PMD, as demonstrated by the superior reduction of M1 lung CFU counts by the BPaMZ regimen compared to that of BMZ in experiment 1 and the superior prevention of relapse by BPaMZ after 1.5 months of treatment in experiment 2. Notably, the addition of PMD to BMZ significantly reduced M1 lung CFU counts but not the proportion of mice relapsing after 1.5 months of treatment in experiment 1, whereas it significantly reduced the proportion of mice relapsing and/or the CFU counts in relapsing mice after 1 and 1.5 months of treatment, but not M1 CFU counts, in experiment 2. This apparent discordance is most likely attributable to the relatively small magnitude of the overall PMD effect in this regimen in this mouse model such that each experiment had only marginal power to detect statistically significant differences.

The present study also confirmed the contribution of PMD to the bactericidal activity of the BPaL regimen observed in prior studies (6, 12) and shows, for the first time, the key contribution of PMD to the sterilizing activity of this regimen. The treatment-shortening effect of including PMD in the BPaL regimen is notably larger than the effect of including it in the BPaMZ regimen in BALB/c mice. The difference in the magnitude of the PMD effect in these 2 regimens likely stems from the sterilizing activity of the respective companion drugs in each regimen in this mouse model. The BMZ combination has powerful sterilizing activity, driven largely by the synergistic activity of BDQ and PZA, that presumably leaves fewer persisting bacilli that are differentially more susceptible to PMD than any other drug in the regimen. The larger treatment-shortening effect of PMD when added to BMZ against the PZA-resistant *pncA* mutant further emphasizes the extent to which the inclusion of PZA, when active, reduces the magnitude of the PMD effect. It also emphasizes the important role PMD may play in the BPaMZ regimen when it is applied to MDR-TB patients, who often harbor PZA-resistant *pncA* mutants that commonly go undetected owing to the challenges of PZA susceptibility testing in low-resource areas.

Prior work has established immunocompromised athymic nude mice as a more stringent model for measuring the sterilizing activity of rifamycin-based regimens and their ability to restrict the emergence of resistance, indicating a beneficial contribution of cell-mediated immunity to the treatment response and to the suppression of drug-resistant mutants, which may be less fit (15, 16). As we predicted from these prior studies (15, 16), we observed slower killing by BPaMZ and BMZ in athymic nude mice than BALB/c mice. These results indicate that the adaptive immune response plays an additive role in bacterial elimination during treatment with these regimens, either through direct immune-mediated killing or by potentiating the activity of one or more drugs in the regimen. Also as expected, the eradication of all cultivable bacteria with BPaMZ required a longer treatment duration in athymic nude mice. Nevertheless, BPaMZ rendered nude mice culture negative with ≤2 months of treatment and prevented relapse in nearly 95% of mice after 2.5 months of treatment. The absence of relapse in nude mice is likely to reflect true sterilization of infection by the regimen, especially considering the rapid demise of 3 of 4 relapsing mice in the BMZ arm, and affirms the intrinsic sterilizing activity of this drug combination. It should be noted that the bactericidal and sterilizing activity of BPaMZ in this experiment was superior to that of a rifapentine-isoniazid-PZA regimen previously evaluated in the same model (15) that is currently being investigated (with the addition of ethambutol) as a 4-month regimen for drug-susceptible TB. Inclusion of PMD significantly reduced lung CFU counts over the first month of treatment and reduced the proportion of mice relapsing, although the effect of PMD on relapse did not reach statistical significance. These results suggest that the contribution of PMD to the regimen will extend to immunocompromised hosts.

To further examine the contribution of PMD to BPaMZ, we used the emerging C3HeB/FeJ mouse model to better mimic the pathophysiological conditions found within caseating human lung lesions (e.g., hypoxia, more neutral pH) (18–20, 22, 24, 27). Prior observations have indicated reduced diffusion of BDQ into the caseous regions of necrotic lung lesions relative to the bordering cellular regions and reduced activity of PZA in caseum with near-neutral pH in this strain (23, 24). On the other hand, PMD appears to diffuse well through caseum and is active under hypoxic conditions (10, 28); thus, it should lend important bactericidal activity to caseous lesions. The high infectious dose and repeated aerosol infection protocol used in experiment 3 resulted in extensive lung involvement with large, often coalescing, caseating granulomas, caseous pneumonia, and, over time, cavitation. The massive bacterial burden and extensive caseation present at the onset of treatment resulted in further pathological progression and death despite the initiation of the highly bactericidal BPaMZ and BMZ regimens. Despite this advanced pulmonary pathology, the inclusion of PMD in the regimen significantly increased the median survival time and bactericidal activity, and the regimen rendered all mice culture negative after 2 months of treatment, with the exception of a single mouse with 1 detectable CFU. It is tempting to speculate that the larger effect of PMD on M1 CFU counts in this model compared to that in BALB/c and nude mice was a result of its superior distribution and/or activity within caseating lesions compared to that of BDQ and PZA. Further studies are warranted to assess the treatment-shortening effect of including PMD in the BPaMZ regimen in this model. Nevertheless, these studies provide further reassurance that the results observed with BPaMZ, and the contribution of PMD specifically, are translatable to human TB.

BDQ is a key bactericidal and sterilizing component of the BPaMZ and BPaL regimens (4, 6). As such, it exerts strong selection pressure, and bactericidal and sterilizing companion drugs are necessary to restrict the selective amplification of spontaneous BDQ-resistant mutants. Previous studies have identified BDQ-resistant mutants selected *in vitro* in mice and in TB patients (13, 29–31). In most cases in which it emerged *in vivo*, resistance was attributable to mutations in the transcriptional repressor *Rv0678* or in the predicted proline aminopeptidase *pepQ*, although the latter mutation target has yet to be confirmed in BDQ-resistant clinical isolates. In the present study, we tracked the selection of BDQ-resistant mutants, and the ability of PMD to prevent such selection, in immunocompromised nude mice in experiment 1, BALB/c mice in experiment 2, and severely diseased C3HeB/FeJ mice in experiment 3. The BPaMZ regimen proved to be quite robust to the emergence of resistance in each model. The only instance in which a BDQ-resistant isolate was observed after BPaMZ treatment was a single mouse in experiment 2 that was originally infected with a PZA-resistant strain, whereas 6 mice received BMZ against the same strain. Likewise, inclusion of PMD in the BPaL regimen significantly reduced the proportion of relapsing mice with BDQ resistance. These findings demonstrate the limited ability of LZD and MXF to prevent the selective amplification of BDQ resistance on their own and suggest that PZA plays an important role in preventing such amplification among phenotypic subpopulations similar to those represented in BALB/c mice.

Interestingly, no selection of BDQ resistance was observed with BPaMZ or BMZ treatment of C3HeB/FeJ mice despite the large bacterial burden and the expected limited contribution of PZA in caseous lesions in this model (24). PMD and MXF partition more into the cellular regions near the perimeter of caseous lesions than acellular caseum, although concentrations in caseum still exceed the MIC (28, 32). MXF accumulates particularly well into foamy macrophages and also accumulates in neutrophils (33), both of which harbor substantial numbers of bacilli at the foamy macrophage-caseum interface. From these cells, it diffuses into adjacent caseum, resulting in greater accumulation in caseum with greater cellularity (32), which tends to correlate with areas of higher bacterial burden. Our results suggest that PMD and MXF are sufficiently effective in the caseum and intracellular compartments, while PZA works primarily in the intracellular compartment, to effectively prevent the selection of BDQ-resistant mutants in the various lesion compartments found in the lungs of severely diseased C3HeB/FeJ mice. Similarly, little BDQ resistance selection was observed in nude mice, and only in a single BMZ-treated mouse, despite evidence of spontaneous resistant mutants present at baseline. At the beginning of treatment, all nude mice sampled harbored *Rv0678* mutant subpopulations and 2 of 5 harbored spontaneous *pepQ* mutants (40%). Consistent with some prior observations (30, 34), frameshift mutations in *Rv0678* caused higher MICs than single-nucleotide polymorphisms in *Rv0678* and *pepQ* mutations. It is noteworthy that the MICs associated with the latter mutants, in particular, hover around the recently proposed critical concentration for BDQ susceptibility testing on 7H11 agar (35) and, hence, may not be recognized as resistant.

In conclusion, PMD contributed significantly to the efficacy of both the BPaMZ and BPaL regimens and reduced the selection of bedaquiline-resistant mutants. These results support further clinical trials to confirm the therapeutic utility of each of these PMD-containing regimens.

## MATERIALS AND METHODS

### Bacterial strains.

M. tuberculosis H37Rv was mouse passaged, frozen in aliquots, and subcultured in Middlebrook 7H9 broth supplemented with 10% oleic acid-albumin-dextrose-catalase (OADC) (Fisher, Pittsburgh, PA) and 0.05% Tween 80 prior to infection. An isogenic PZA-resistant mutant was selected from a mouse infected with the H37Rv strain and treated with PZA monotherapy, and an isolated mutation in *pncA* (A146V) was confirmed by whole-genome sequencing (24). The PZA MIC for this *pncA* mutant is ≥900 μg/ml (versus 150 μg/ml for the parent H37Rv strain) by 7H9 broth dilution at pH 6.8. An isogenic PMD-resistant mutant was selected from a mouse infected with the H37Rv strain and treated with PMD monotherapy, and an isolated mutation in *ddn* (M1T) was confirmed by whole-genome sequencing. The pretomanid MIC for this *ddn* mutant is ≥16 μg/ml (versus 0.06 to 0.125 μg/ml for the parent H37Rv strain) by 7H9 broth dilution. M. tuberculosis HN878 was used as frozen stocks prepared from a log-phase culture in Middlebrook 7H9 broth after mouse passage and was diluted in phosphate-buffered saline (PBS) before infection.

### Aerosol infection with M. tuberculosis.

All animal procedures were approved by the Animal Care and Use Committee of Johns Hopkins University. Six-week-old female BALB/c mice and immunodeficient athymic CD-1 nude mice (Charles River Laboratories, Wilmington, MA) were infected with M. tuberculosis H37Rv or the isogenic *pncA* or *ddn* mutant, using the inhalation exposure system (Glas-Col, Terre Haute, IN) and a fresh log-phase broth culture (optical density at 600 nm, 0.8 to 1.0), with the goal of implanting 4 log_10_ CFU in the lungs of each mouse (4). Four or five mice were humanely killed 1 day after infection (D−13) and on the day of treatment initiation (D0) to determine the number of bacteria implanted in the lungs and at the start of treatment, respectively.

Female C3HeB/FeJ mice (Jackson Laboratory, Bar Harbor, ME), 10 weeks old, were aerosol infected with M. tuberculosis HN878 on two occasions spaced 10 days apart (with mice divided into 2 runs per occasion) in a repeated infection protocol intended to promote more advanced caseating lung lesions. On each occasion, a frozen stock culture was thawed and diluted with the intention to implant approximately 200 CFU per run. Treatment started at 4 weeks after the first infection (W−4). Six and nine mice (2 or 3 mice per run) were sacrificed for lung CFU counts at W−4 and D0 to determine the number of CFU implanted and the number present at the start of treatment, respectively.

### Chemotherapy.

Drugs were prepared as previously described and administered once daily, 5 days per week, by gavage (11). The drug doses (in milligrams per kilogram of body weight) were the following: BDQ, 25; PMD, 100; MXF, 100; PZA, 150; LZD, 100 (4, 6). BDQ was formulated for oral administration in an acidified 20% hydroxypropyl-β-cyclodextrin solution as previously described (11). PMD was suspended in a cyclodextrin micelle (CM-2) formulation containing 10% hydroxypropyl-β-cyclodextrin (Sigma) and 10% lecithin (ICN Pharmaceuticals Inc., Aurora, OH) (36). MXF and PZA were dissolved in distilled water. LZD was suspended in a solution composed of 5% polyethylene glycol 200 (PEG 200; Sigma) and 95% methylcellulose (0.5%; Fisher, Suwanee, GA) in distilled water (11). BDQ and PMD were prepared separately and administered together after mixing just prior to administration. MXF+PZA or LZD was administered at least 4 h later.

### Assessment of treatment efficacy.

Efficacy was assessed on the basis of lung CFU counts at selected time points during treatment (a measure of bactericidal activity) and the proportion of mice with culture-positive relapse after treatment completion (a measure of sterilizing activity). Lung homogenates were plated in serial 10-fold dilutions onto selective 7H11 agar plates supplemented with 0.4% activated charcoal to reduce carryover effects (11) and incubated for 6 weeks before determining final CFU counts.

### Evaluation of resistance selection.

Aliquots representing one-fifth of the lung homogenates were plated directly onto selective 7H11 agar containing 0.06 (2× MIC) or 0.125 μg/ml (4× MIC) of BDQ or 2 μg/ml (16× to 32× MIC) of PMD to quantify the proportion of CFU resistant to either drug at selected time points before, during, and after treatment. These concentrations reliably select for *Rv0678* and *pepQ* mutants resistant to BDQ or *ddn*, *fgd,* and F420 biosynthesis mutants resistant to PMD (4, 11, 13, 37). Colonies isolated on BDQ-containing plates were selected and analyzed by PCR and DNA sequencing of the *Rv0678*, *pepQ,* and *atpE* genes, as described previously (13). The MICs of bedaquiline-resistant isolates and H37Rv were determined using the broth macrodilution method with doubling concentrations of bedaquiline from 0.03 to 2 μg/ml (13). Briefly, tubes containing 2.5 ml of 7H9 broth plus OADC without Tween 80 with the above-mentioned concentrations of bedaquiline were inoculated with 10^5^ CFU of log-phase culture of H37Rv or BDQ-resistant isolates. The MIC was defined as the lowest concentration that prevented visible growth after 14 days of incubation at 37°C.

### 16S rRNA sequencing for identification of bacteria.

Genomic DNA was extracted from bacterial colonies using the cetyltrimethylammonium bromide (CTAB) method. 16S rRNA was amplified with primers 16S-F (5′-AGAGTTTGATCCTGGCTCAG-3′) and 16S-R (5′-ACGGGCGGTGTCTACAA-3′), targeting positions 11 to 1399 of the 16S rRNA gene (38). PCR products were purified with QIAquick PCR purification kits (Qiagen, Germany), mixed with primers, and then sequenced (GeneWiz Inc.). Species identification was performed using BLAST search (GenBank database sequences) with sequence data.

### Pathology.

Two mice in each group at each time point were chosen for histopathology. Lungs were collected after systemic perfusion with PBS under deep anesthesia, fixed in 4% paraformaldehyde, embedded in paraffin, and cut to 5-μm thickness (20). Subsequent tissue sections were mounted on glass slides, deparaffinized, and stained with hematoxylin-eosin (H&E) and Ziehl-Neelsen (AFB). Sections were scanned and visualized using Aperio Imagescope software (Leica Biosystems, Nussloch, Germany), allowing a wide variety of magnifications (19). Low-power views of an entire lung section and higher-power views of individual granulomas or cavitary lesions from mice in each group were shown.

### Statistical analysis.

Lung CFU counts (*x*) were log transformed (as *x* + 1) before analysis, and mean and median CFU counts were compared using Student's *t* test and Kruskal-Wallis tests, respectively. The proportions of mice relapsing were compared using Fisher’s exact test. Survival analyses were performed using the Kaplan-Meier method (20), and the log-rank test was used to compare the observed differences in survival. All analyses were performed with GraphPad Prism, version 5 (GraphPad, San Diego, CA).

## Supplementary Material

Supplemental file 1
