# Supplementary material for: Contribution of Pretomanid to Novel Regimens Containing Bedaquiline with either Linezolid or Moxifloxacin and Pyrazinamide in Murine Models of Tuberculosis
Source: Antimicrob Agents Chemother. 2019 Apr 25;63(5):e00021-19. doi: 10.1128/AAC.00021-19 (PMC6496099; doi:10.1128/AAC.00021-19)
Supplement: Supplemental file 1 [file AAC.00021-19-s0001.pdf]

Supplementary information

Table S1. Scheme of Experiment 1 to evaluate the contribution of PMD to the BPaMZ regimen in BALB/c and nude mice

Mice	Regimen	Time point* and No. of mice sacrificed						
		D-13	D0	M1	M2	M1.5(+3)	M2(+3)	M2.5(+3)
BALB/c	BPaMZ	5	4	5		15	16	
	BMZ			5		15	15	
Nude	BPaMZ	5	5	10	10			18
	BMZ			10	10			16

*(+3) indicates mice held for an additional 3 months beyond the completion of treatment (e.g., M2 (+3) indicates mice were treated for 2 months before being held without treatment for an additional 3 months prior to sacrifice)

Table S2. Mutations observed in *Mycobacterium tuberculosis* colonies isolated from nude mice on bedaquiline-containing plates and the corresponding MIC

Mouse No.	Sample No.	*Rv0678*	*pepQ*	*atpE*	MIC (µg/ml)
1 (D0)	1	WT	g415a (E139K)		0.25
	2	WT	g415a (E139K)		
	3	WT	g415a (E139K)		
	4	WT	g415a (E139K)		
	5	gt133-134 deletion (45 codon shift)	WT		0.25
2 (D0)	6	gt314-315 deletion (105 codon shift)	WT		0.5
	7	t221c (L74P)	WT		0.5
	**8***	IS6110 insertion nt 349 (117 codon shift)	WT	WT	0.5
3 (D0)	9	t425 deletion (142 codon shift)	WT		0.5
	10	gc71 insertion (24 codon shift)	WT		0.5
	11	WT	g812 insertion (271 codon shift)		0.25
	12	IS6110 insertion nt 94 (32 codon shift)	WT	WT	0.5
4 (D0)	13	c268t (R90C)	WT		0.25
	14	t461c (L154P)	WT		0.5
	15	c466 insertion (156 codon shift)	WT		0.5
	**16***	g259 insertion (87 codon shift)	WT	WT	0.5
	17	IS6110 insertion nt 65 (22 codon shift)	WT	WT	0.5
5 (D0)	18	g126a (W42stop)	WT		0.5
	19	IS6110 insertion nt 334 (112 codon shift)	WT	WT	0.5
	20	IS6110 insertion nt 334 (112 codon shift)	WT	WT	0.5
	21	IS6110 insertion nt 334 (112 codon shift)	WT	WT	0.5
	22	g362t (G121V)	WT		0.25
Relapse	BMZ-1 (W10+6)	c313t (R105C)	WT		
	BMZ-8 (W10+12)	WT	g896t (G299V)		0.25

*indicates that BDQ-resistant strains were sequenced by whole genome sequencing

Table S3. Scheme of Experiment 2 to evaluate the contribution of PMD in the BPaL and BPaMZ regimens against *M. tuberculosis* H37Rv (wild-type) and a *pncA* mutant (in italics)

Regimen	Time point* and No. of mice								
	**D-14**	**D0**	**M1**	**M2**	**M1 (+3)**	**M1.5 (+3)**	**M2 (+3)**	**M3 (+3)**	**M4 (+3)**
Untreated	4	6							
BL			5	5			15	15	15
BPaL			5	5			15	15	15
BMZ			5		15	15	15		
BPaMZ			5		15	15	15		
Untreated	*2*	*3*							
BMZ			*5*	*5*			*15*	*20*	
BPaMZ			*5*	*5*			*15*	*20*	

*(+3) indicates mice held for an additional 3 months beyond the completion of treatment

Table S4. Scheme of Experiment 2 to evaluate the contribution of PMD in the BPaL and BPaMZ regimens against a *ddn* mutant

Regimen	Time point* and No. of mice						
	**D-14**	**D0**	**M1**	**M2**	**M1(+3)**	**M2 (+3)**	**M3(+3)**
Untreated	4	6					
Pa$_{50}$			5	5			
Pa$_{100}$			5	5			
BL			5	5			15
BPa$_{50}$L			5	5			15
BPa$_{100}$L			5	5			15
BMZ			5	5	15	15	
BPa$_{50}$MZ			5	5	15	15	
BPa$_{100}$MZ			5	5	15	15	

*(+3) indicates mice held for an additional 3 months beyond the completion of treatment

†number in subscript indicates the drug dose in mg per kg of body weight

Table S5. Scheme of Experiment 3 to evaluate the contribution of PMD in the BPaMZ regimen in C3HeB/FeJ mice

**Regimen**	**Time point and No. of mice**				
	**W-4**	**D0**	**M1**	**M2**	**M3**
Untreated	12	10	10		
BMZ			10	10	10
BPaMZ			10	10	10

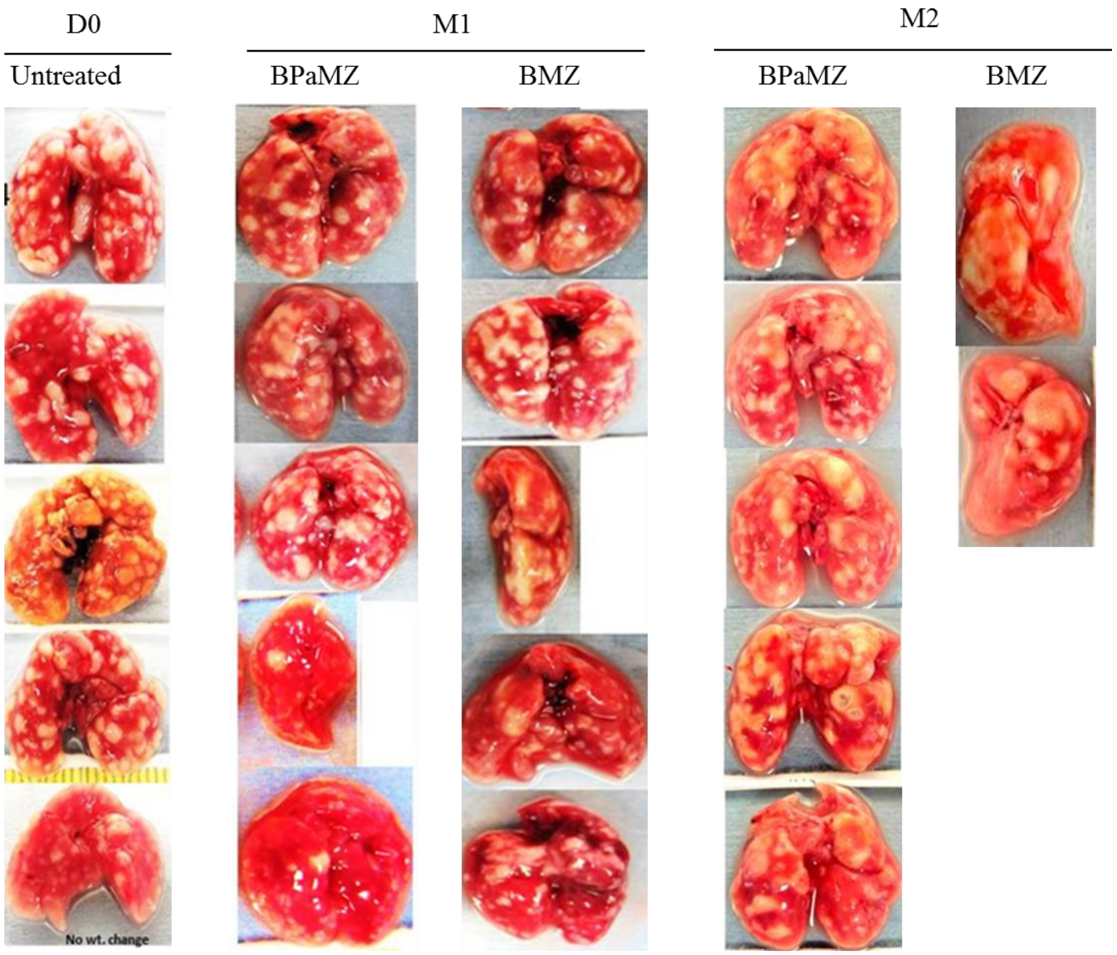

Figure S1. Gross lung pathology observed in C3HeB/FeJ mice before and during treatment with BPaMZ or BMZ beginning 4 weeks post-infection with *M. tuberculosis* HN878.
